# Neonatal Sepsis due to Coagulase-Negative Staphylococci

**DOI:** 10.1155/2013/586076

**Published:** 2013-05-22

**Authors:** Elizabeth A. Marchant, Guilaine K. Boyce, Manish Sadarangani, Pascal M. Lavoie

**Affiliations:** ^1^Child and Family Research Institute, 4th Floor, Translational Research Building, 950 West 28th Avenue, Vancouver, BC, Canada V5Z 4H4; ^2^Department of Medicine, University of British Columbia, Vancouver, BC, Canada V6T 1ZA; ^3^Department of Pediatrics, University of British Columbia, Vancouver, BC, Canada V6T 1ZA; ^4^Department of Pediatrics, Children's Hospital, University of Oxford, Oxford OX3 9DU, UK

## Abstract

Neonates, especially those born prematurely, are at high risk of morbidity and mortality from sepsis. Multiple factors, including prematurity, invasive life-saving medical interventions, and immaturity of the innate immune system, put these infants at greater risk of developing infection. Although advanced neonatal care enables us to save even the most preterm neonates, the very interventions sustaining those who are hospitalized concurrently expose them to serious infections due to common nosocomial pathogens, particularly coagulase-negative staphylococci bacteria (CoNS). Moreover, the health burden from infection in these infants remains unacceptably high despite continuing efforts. In this paper, we review the epidemiology, immunological risk factors, diagnosis, prevention, treatment, and outcomes of neonatal infection due to the predominant neonatal pathogen CoNS.

## 1. Epidemiology of Neonatal Sepsis

Neonatal sepsis is defined as infection in the first 28 days of life, or up to 4 weeks after the expected due date for preterm infants [1]. Epidemiologists defined two types of infections in neonates: early-onset neonatal sepsis (EONS), which manifests in the first 72 hours of life (up to 7 days) and late-onset neonatal sepsis (LONS), whose incidence peaks in the 2nd to 3rd week of postnatal life [1]. The mortality from neonatal sepsis has dramatically decreased over the last century, because of medical advances. In the preantibiotic era (<1940), the case fatality rate of neonatal sepsis was extremely high, exceeding 80% [2]. By the late 1960s, the introduction of antibiotics and the development of modern perinatal care had lowered this case fatality rate to less than 20% overall [2]. The composition of pathogens causing neonatal sepsis has also changed dramatically over the last century [2–6]. In the early 1930s, *Streptococcus pneumoniae* and group A streptococci were responsible for almost half of the cases of LONS [2, 3]. By the 1960s, gram-negative bacilli had become major pathogens [3], along with the emergence of group B streptococci (GBS) as a predominant cause of EONS [2]. In North America, gram-positive organisms account for the majority of neonatal sepsis cases (up to 70%). Sepsis due to gram-negative organisms (~15 to 20%) and fungi (~10%) is less common, and polymicrobial bloodstream infections contribute to less than 15% of cases [2, 7, 8]. Coagulase-negative staphylococci (CoNS) are the major pathogen involved in LONS, particularly in infants born at a lower gestational age. According to more recent data from the National Institutes of Child Health and Development (NICHD), infection-related mortality in very low-birth-weight (VLBW) infants (birth weight < 1500 grams) averages 10% [7] but can reach 40% depending on the pathogen involved [9–11]. Preterm neonates have a high risk of developing neonatal infections, resulting in high mortality and serious long-term morbidities [5, 7, 12]. In North America, it is estimated that each episode of sepsis prolongs the duration of a neonate's hospital stay by about 2 weeks, resulting in an incremental cost of USD$25,000 per episode [13]. In a more recent study, authors estimated that nosocomial bloodstream infections increase the neonatal hospitalization cost for VLBW infants in the lowest birth weight group (401–750 grams) by 26%, and that of the highest birth weight group (1251–1500 grams) by 80% [14]. This study also estimated that the duration of hospital stay increased by four to seven days in all VLBW categories with a nosocomial bloodstream infection [14]. 

### 1.1. Burden of Neonatal Sepsis in Developing Countries

In developed countries, advances in medical care have enabled a greater proportion of premature infants to survive, albeit with an increased risk of infection [15]. However, because the greatest burden of neonatal sepsis falls on low-resource developing countries, the global economic impact is difficult to estimate [16]. Globally, infections still cause an estimated 1.6 million neonatal deaths annually, representing 40% of all neonatal deaths [16–18]. About 12% of children are born prematurely worldwide, including about 2% of VLBW. Together, prematurity and neonatal infections account for the greatest burden of neonatal deaths overall [16]. The limited access to medical resources combined with geographical comorbidities (e.g., severe malnutrition) can lead to mortality from neonatal sepsis remaining unacceptably high in developing countries [16].

## 2. Pathogenesis

Within the first week of life, neonates become rapidly colonized by microorganisms originating from the environment [19–22]. During this period, the risk of CoNS infection increases substantially with the use of central venous catheters (CVC), mechanical ventilation, and parenteral nutrition, and with exposure to other invasive skin- or mucosa-breaching procedures [8, 15, 23–28]. CoNS are common inhabitants of the skin and mucous membranes; although a small proportion of neonates acquire CoNS by vertical transmission, acquisition primarily occurs horizontally [22, 29]. Consequently, infants admitted to a hospital obtain most of their microorganisms from the hospital environment, their parents, and staff [30, 31]. Transmission via the hands of hospital staff can lead to endemic strains circulating for extended periods [29, 32–35]. Because CoNS is a ubiquitous skin commensal, authors have assumed that colonizations of the skin and of indwelling catheters are important sources of sepsis [34, 36]. However, recent studies suggest that epithelial loci other than the skin, such as the nares, may be important access points of infection [34, 36]. Antibiotic resistance in skin-residing strains has been found to be low at birth but to increase rapidly during the first week of hospitalization [37]. Selective pressure as a result of perinatal antibiotic exposure, therefore, is an additional major factor influencing the spectrum and antibiotic resistance pattern of microorganisms isolated from neonates.

### 2.1. Host Immunological Factors

Some components of the immune response are particularly important in preventing sepsis due to CoNS (reviewed in [38]). The immune system is traditionally described in terms of the innate and the adaptive immune systems. The innate immune system is responsible for the “naïve,” more rapid, first-line response to infection. At birth, the neonate's own adaptive immune system is largely uneducated. To protect against infection, neonates must therefore rely heavily on innate immune responses and on passive adaptive immune mechanisms acquired from the mother (e.g., transplacental transfer of antibodies), which are deficient in preterm neonates [39–43]. Specific host innate immune factors have been studied in the context of neonatal CoNS infections: mucosal barriers, including antimicrobial peptides (AMP), cells (neutrophils), and pattern recognition receptors (PRR, e.g., Toll-like receptors), as detailed below.

#### 2.1.1. Mucosal Barriers

 The outermost layer of the skin (*stratum corneum*) acts as a physical barrier and first line of defense against bacterial invasion. The skin secretes AMP, which are early-response factors creating a microbicidal shield particularly effective against CoNS [43–48]. In preterm neonates, the immature *stratum corneum *only fully matures at one to two weeks after birth [42, 43, 46]. The vernix caseosa, a waxy coating on neonates' skin, provides additional antimicrobial protection in mature neonates. It is mainly formed during the last trimester of gestation, leaving extremely premature neonates far more vulnerable to infection [49–51]. Immunity against CoNS is also limited in other mucosal surfaces in preterm neonates, for example, because of a thinner glycocalyx layer coating the intestinal epithelium [52, 53], lower secretory IgA [53], and reduced AMP production by Paneth cells [54–56]. Necrotizing enterocolitis (NEC) is a progressive ischemic necrosis of the neonatal intestine that occurs in preterm infants [57]. The cause of NEC is unclear but is believed to develop as a result of gut injury, with a key role for bacteria in its pathogenesis [57–59]. Frequent isolation of enterotoxin-producing CoNS from the intestinal flora of infants with NEC has led authors to propose that overgrowth of CoNS plays a role in this complication [60–62]. A poor barrier function and an overall immaturity of the premature gastrointestinal immune system [63, 64] contribute largely to the development of NEC, possibly by favoring bacterial overgrowth and translocation [57, 63, 65]. 

#### 2.1.2. Cells

 Neutrophils also play a major role in protection against neonatal sepsis, including CoNS, as first-responder leukocytes in the blood [66–69]. Certain characteristics of neonatal neutrophils have been proposed as mechanisms of increased susceptibility to CoNS sepsis [70]: their relatively inefficient recruitment and extravasation to the site of infection [69]; their reduced bacterial killing capacity, in part due to the failure to upregulate their oxidative burst response [71]; and the reduced ability of neonatal neutrophils to form “extracellular traps” [50]. 

#### 2.1.3. Pattern Recognition Receptors

 PRR detect the presence of microorganisms in the tissue through the recognition of conserved molecular structures specific to microbes (known as pathogen-associated molecular patterns: PAMP). To date, the best characterized PRR are the Toll-like receptors (TLR), which include ten receptors in humans [72–75]. Recent studies in mice have suggested that Toll-like receptor 2 (TLR2), an extracellular member of the TLR family, plays an important role in the immune recognition of CoNS [76]. Additionally, *S. epidermidis* induces an upregulation of TLR2 and MyD88, and a systemic increase in proinflammatory cytokines (e.g., interleukin (IL)-6) [77]. As inflammatory stimuli, the PAMP produced by the gram-positive CoNS are less potent than PAMP expressed at the surface of gram-negative bacteria (e.g., lipopolysaccharide, LPS). However, the most prevalent clinical isolate of CoNS, *S. epidermidis*, is known to produce a complex of bacterial peptides called phenol-soluble modulins, which induce a considerable proinflammatory response through TLR2 [78–80]. Interestingly, activation of TLR2 by a yet unidentified product of *S. epidermidis *triggers the enhanced production of the human AMP family of *β*-defensins from keratinocytes and underscores a potential role of AMP in the control of staphylococcal infections [81, 82]. Reliance on TLR-induced CoNS immunity has important implications, since preterm neonates exhibit marked defects in TLR signaling cascades and cytokine responses [39, 83]. Indeed, monocytes of premature neonates display a gestational age-dependent reduction in TLR-induced production of proinflammatory cytokines [84], whereas other monocyte functions related to phagocytosis and intracellular bacterial killing develop earlier, well before 30 weeks of gestation [85].

### 2.2. Bacterial Virulence Factors

CoNS lacks several of the virulence factors shared with the closely related species *S. aureus* [31]. Compared with *S. aureus, S. epidermidis* produces lower levels of cytolytic toxins [86]. Therefore, *S. epidermidis* must rely on other mechanisms, such as biofilms and the anionic polymer poly-*γ*-DL-glutamic acid (PGA) to evade hosts' immune responses.

Biofilm formation serves as the primary mode of immune evasion of CoNS [87]. These multilayered bacterial aggregates strongly adhere to inanimate objects such as indwelling medical devices. CoNS are particularly adept at biofilm formation, and this capacity is a key mechanism of their pathogenesis, particularly in relation to catheter-related infections [88, 89]. Biofilms act as nonselective physical barriers that obstruct antibiotic diffusion and hinder the cellular and humoral host immune responses [86, 90–93]. In addition, biofilms provide protection from antimicrobial therapy [30, 31, 94, 95]. Poly-*N-*acetylglucosamine surface polysaccharide, also termed polysaccharide intercellular adhesin (PIA), is crucial in facilitating cellular aggregation during biofilm formation and is the most extensively studied biofilm molecule [31, 90]. In rat models, PIA defective mutants have been shown to exhibit decreased virulence [31]. Lack of PIA in *S. epidermidis *results in mutants susceptible to phagocytosis and killing by human neutrophils as well as enhanced AMP susceptibility [92]. Additionally, the expression of an ATP-binding cassette transporter allows for the export of AMP out of the bacterial cell, thus contributing to AMP resistance [86, 96]. Other components help CoNS evade immune defenses; for example, a glutamyl endopeptidase from *S. epidermidis* is expressed specifically in biofilms and degrades the complement-derived chemoattractant C35 [31]. 

The secreted anionic extracellular polymer PGA also plays an important role in immune evasion of *S. epidermidis* [91]. However, PGA is not specific to *S. epidermidis*, and is also secreted by other staphylococcal species and *Bacillus* strains [91, 97]. PGA appears to play an important role in the persistence of *S. epidermidis* colonization on medical devices [91]. Moreover, PGA contributes to resistance against phagocytosis and microbicidal action of AMP like LL-37 and human *β*-defensin 3, as demonstrated by increased susceptibility to neutrophils and AMP activity; however, the precise mechanisms of this PGA-mediated resistance remain unclear [91]. To avoid antistaphylococcal human AMP, *S. epidermidis* is also equipped with resistance mechanisms such as the Aps (antimicrobial peptide sensing) system and the AMP-degrading protease SepA [86, 96].

Finally, bacteria have multiple creative antibiotic resistance mechanisms, including modification of target structures (e.g., altered penicillin-binding proteins in staphylococci) and production of antibiotic-inactivating enzymes (e.g., beta-lactamases to hydrolyze penicillins, cephalosporins and/or carbapenems). Genes encoding proteins responsible for these mechanisms often reside on mobile genetic elements, enabling transfer of resistance between bacteria of the same or different species. In a recent study, authors proposed that CoNS may be a significant reservoir of methicillin resistance genes that can be transferred horizontally to other common related neonatal pathogens such as *S. aureus *[98].

## 3. Diagnosis

Neonatal sepsis is clinically diagnosed by a combination of clinical signs, nonspecific laboratory tests and microbiologically confirmed by detection of bacteria in blood by culture. Clinical signs of sepsis in neonates are usually nonspecific and often inconspicuous. They include the presence of fever or hypothermia (in the preterm neonate, this is more commonly seen as a general disturbance in thermoregulation); lethargy; poor feeding; respiratory distress or apnea; pallor; jaundice; tachycardia or bradycardia; hypotension; disturbances in gastrointestinal function (diarrhea, bloody stools, abdominal distention, and ileus); and thrombocytopenia [30, 31, 99, 100]. With CoNS, such clinical signs are often more subtle because of the low virulence of these organisms. However more serious, often persistent illness due to more virulent strains can occur in a considerable minority of cases, in association with severe thrombocytopenia [101]. 

The gold standard for diagnosis of neonatal sepsis remains blood culture. However, in many situations this test is fraught with practical problems, including the small blood volumes obtainable, especially in the smallest of preterm neonates. Indeed, this volume is often below the recommended 1 mL lower limit of detection, leading to a high proportion of false negative test results [102–105]. Conversely, the nonspecific nature of clinical signs in neonates probably leads to frequent overuse of broad-spectrum antibiotics with the potential to select for resistant bacteria and fungi, especially in preterm neonates. Therefore, there is a great need for better rapid diagnostic tests to differentiate infants with sepsis from those who are sick from other causes.

Hematological indices (e.g., numbers of white blood cells, neutrophils, platelets) [106] and biochemical markers of inflammation, such as C-reactive protein [107], and procalcitonin [108] are routinely used in clinical practice and can aid in the diagnosis of neonatal sepsis. This is particularly useful in cases of persisting clinical symptoms and in the absence of a confirmatory positive blood culture, or in situations where localized sources of infection are being considered [105]. Furthermore, the abundance of CoNS as a natural skin commensal often leads to blood culture contamination and a subsequent overestimation of neonatal sepsis cases [109, 110]. 

CVC, which are often used in smallest preterm neonates, provide a sanctuary for CoNS, leading to persistence of an infection. A number of methods to determine if the CVC is the source of an infection have been suggested, including observing a positive culture from the CVC but not from a peripheral site [111, 112] and reduced “time to positivity” of a CVC culture (as opposed to a peripheral site). A higher bacterial load in the CVC [113, 114] and a three- to fivefold differential magnitude of colony-forming units between a quantitative CVC and peripheral culture are indicative of a CVC as the primary focus of infection [99, 115]. However, these methods are impractical when applied to neonates. The small lumen size of the CVC makes removal of blood, and therefore CVC culture, impossible in most cases. Furthermore, any comparison of CVC and peripheral cultures would rely on identical sample volumes from both sites being taken and processed at exactly the same time, which is often not feasible.

In the future, new diagnostic technologies involving microfluidics may considerably reduce the amount of blood volumes required for diagnosis [116]. At present, the relatively high cost of this technique limits its routine use in the clinical setting [117]. In some instances, polymerase chain reaction (PCR) can be useful to characterize subspecies [118, 119]. Adjunctive use of nucleic acid-based technologies with blood cultures can facilitate a faster diagnostic turnaround time and easier antibiotic susceptibility profile identification. Molecular typing techniques, such as pulsed field gel electrophoresis and multilocus sequence typing [99], are also useful in subspecies differentiation [120]. PCR-based diagnostic methods may be most useful clinically in the short term by providing clinicians with the ability to detect the presence of genetic markers of antibiotic resistance [118].

## 4. Prevention and Treatment

### 4.1. Prevention

 In the hospital setting, the mainstay of prevention against neonatal sepsis includes strict hand-washing practices; careful aseptic procedures in the management of intravenous lines; skin care; judicious use of antibiotics; promoting early enteral (as opposed to parenteral) nutrition, preferably using breast milk (i.e., to enhance the infant's own gastrointestinal immune defenses); and minimizing invasive interventions (e.g., prompt removal of CVCs, reducing mechanical ventilation) [7, 121–123]. Hand washing is a widely accepted and cost-effective measure to decrease the occurrence of nosocomial infections including CoNS [15, 124–127]; yet universal compliance is difficult to achieve [3]. Minimizing the indwelling time and number of CVCs decreases the risk of CoNS and other pathogens of LONS [15, 128]. In some studies, more than half of all cases of CoNS sepsis occurred while indwelling CVCs were in place [129]. The number of central lines experienced by the neonate from birth, rather than the duration of insertion, was an important predictor of CoNS sepsis [28]. Some authors have proposed the use of prophylactic antibiotics immediately before and for 12 hours after removal of a CVC in preterm neonates [129]. 

Clinical trials of vancomycin added to parenteral nutrition solutions have demonstrated decreases in the incidence of CoNS sepsis in preterm neonates [130, 131], without reduction in mortality or duration of hospital stay [132]. Others have proposed using antibiotic-coated devices for CVC [133–135]. However, these measures carry a risk of increasing antimicrobial resistance and have not been universally adopted. Antimicrobial “locks,” that is, leaving a microbicidal substance within the catheters in between administration of other drugs represents another proposed solution to decrease bacterial colonization. Antiseptics (e.g., alcohol, taurolidine), anticoagulants (e.g., heparin, EDTA), and antibiotics (e.g., vancomycin, rifamycin) have all been studied [105, 136–139]. Two studies reported a reduction in catheter-related sepsis in critically ill neonates through the use of either fusidic acid and heparin, or vancomycin locks [140, 141]. The benefit of antibiotic lock over prophylactic antibiotic administration is the avoidance of systemic effects of antibiotics in the patient, since the solution remains within the catheter. A similar measure incorporates antiseptic-impregnated catheters to decrease cutaneous bacterial load and catheter colonization [134, 135, 139, 142]. However, clinical experience with these methods is very limited in VLBW infants. In the absence of more definitive evidence, the standard of care is to use strict hand hygiene and skin antisepsis protocols prior to, during, and after catheter insertion [8, 143]. 

### 4.2. Treatment

The subtle, nonspecific nature of clinical signs and the rapid progression of neonatal sepsis make prompt diagnosis and antibiotic treatment crucial. Any delay in antimicrobial therapy places a neonate with sepsis at greater risk of mortality. Empirical antibiotic therapy should be based on knowledge of local epidemiology and antibiotic resistance patterns of neonatal sepsis, since geographic variation can be influential. Because colonization of infants with CoNS is unusual in the first 48 hours after birth, the preferred empirical treatment of EONS is mainly based on the use of ampicillin and gentamicin to cover more predominant GBS and gram-negative bacilli, and, to a lesser extent, *L. monocytogenes*. For LONS, administration of antistaphylococcal penicillin (e.g., oxacillin) or an alternative agent such as vancomycin is indicated. The advantage of a penicillin is the low toxicity and potent *in vivo* bactericidal activity, even in difficult infections such as endocarditis [30, 31]. In areas with widespread beta-lactam resistance in CoNS and/or a high prevalence of methicillin-resistant *S. aureus*, vancomycin is often preferred [144]. Although not as bactericidal as oxacillin, little resistance has been reported to vancomycin. Considerable rates of gram-negative organisms in LONS dictate that empirical treatment cannot consist solely of antistaphylococcal antibiotics. Therefore, aminoglycosides are frequently used in addition, as in EONS, and may have a synergistic antistaphylococcal effect when administered with penicillins and vancomycin, although the *in vivo* significance of this is not entirely clear [145–147]. Linezolid, another class of antibiotics, possesses potent antistaphylococcal activity comparable to that of vancomycin, with little reported resistance [148, 149]. Once culture results are available, antibiotics can be modified to specifically target the isolated pathogen according to the results of susceptibility testing.

The presence of a CVC or other indwelling foreign material is highly associated with persistence of infection despite appropriate antibiotic therapy, because of biofilm formation. *In vivo* antibiotic action is also antagonized by the neutralization of pharmaceuticals like vancomycin by the polysaccharides of CoNS biofilms [150]. In addition, the low metabolic activity of biofilms limits the activity of many antibiotics which require rapid metabolism of growing bacteria to exert their microbicidal effect [151]. Antibiotic resistance and biofilm formation are among selective factors for the persistence of endemic nosocomial strains and probably contribute to the predominance of *S. epidermidis* and *S. haemolyticus* as clinical isolates on NICU infants [37, 100, 152]. In such cases, it may be imperative to remove the CVC. 

## 5. Long-Term Sequelae

Multiple studies show that neonatal sepsis has major long-term neurodevelopmental consequences in survivors, particularly in preterm infants [153]. In modern intensive care, about half of extremely preterm neonates born at 24 weeks' gestation and the majority of neonates over 25 weeks' gestation generally survive [154]. The risk of such morbidity in extremely premature neonates is inversely proportional to their gestational age [4, 155–158]. In VLBW infants, neonatal sepsis dramatically increases the long-term risk of motor, cognitive, neurosensory and visual impairments [157–159]. The risk of adverse neurodevelopmental outcome in VLBW neonates with sepsis is further increased with other comorbidities such as bronchopulmonary dysplasia [157, 160]. This increased risk of neurodevelopmental impairment in preterm infants with sepsis has several reasons, including a high risk of meningitis; heightened adverse effect of sepsis-associated cardiovascular instability during a vulnerable period for the developing brain; and increased neurotoxic effects of inflammatory mediators [153]. Surprisingly, the risk of adverse neurodevelopmental outcome in VLBW infants surviving from neonatal sepsis does not appear to depend on the infecting organism [157], although in some studies extremely premature infants who experienced sepsis had a greater risk of a hearing impairment when the infection involved gram-negative, fungal, or combined infections [157]. 

## 6. Future Therapies

Despite limited natural antibody immune protection in preterm neonates, meta-analyses of intravenous immunoglobulin administration have so far failed to demonstrate sufficient therapeutic benefits [31, 161–163]. Other immunomodulatory therapies designed to improve neonatal immune deficits, such as granulocyte transfusions, or administration of granulocyte-macrophage colony stimulating factor which increases neutrophils and enhances their antimicrobial activity, have also not yet translated into concrete benefits in clinical trials [161]. Finally, lactoferrin, an antimicrobial glycoprotein that sequesters iron, may be useful in reducing the incidence of late-onset sepsis in low-birth-weight neonates [164]. The future of antistaphylococcal immunotherapy and immunoprophylaxis requires more research. This may require a combined use of adjunctive immunomodulatory treatments to enhance the innate immune system of neonates while disabling virulence factors that enable resistance to conventional antibiotic treatment of CoNS. 

## 7. Conclusion

The 20th century saw CoNS emerge as the foremost pathogen of neonatal sepsis in developed countries. VLBW neonates contribute disproportionately to CoNS-related morbidity and mortality, in stark contrast to their full-term counterparts who usually suffer milder symptoms. Several reasons make prematurity the single most important factor for neonatal sepsis: innate immunological deficiencies; prolonged stays in the NICU; and, notably, the higher use of indispensable but invasive medical interventions in these developmentally immature neonates. Advances in medical technology have dramatically increased the survival rate of premature neonates. This corresponds to a growing burden of both short- and long-term problems associated with neonatal sepsis. Effective prophylactic measures, prompt and accurate diagnoses, and subsequent administration of targeted therapy are vital to curb the excessive burden of disease that CoNS infection imposes upon this highly vulnerable age group.
